# *De Novo* Small Supernumerary Marker Chromosomes Arising From Partial Trisomy Rescue

**DOI:** 10.3389/fgene.2020.00132

**Published:** 2020-02-27

**Authors:** Keiko Matsubara, Kaede Yanagida, Toshiro Nagai, Masayo Kagami, Maki Fukami

**Affiliations:** ^1^ Department of Molecular Endocrinology, National Research Institute for Child Health and Development, Tokyo, Japan; ^2^ MK Clinic, Miyakonojo, Japan; ^3^ Nakagawanosato Ryoiku Center, Saitama, Japan

**Keywords:** chromothripsis, embryo, genomic rearrangement, micronucleus, supernumerary chromosome, uniparental disomy, U-type exchange

## Abstract

Small supernumerary marker chromosomes (SMCs) are rare cytogenetic abnormalities. *De novo* small SMCs, particularly those combined with uniparental disomy (UPD), are assumed to result from incomplete trisomy rescue. Recently, a one-off cellular event designated as chromothripsis was reported as a mechanism for trisomy rescue in micronuclei. This *Perspective* article aims to highlight a possible association among trisomy rescue, chromothripsis, and SMCs. We propose that chromothripsis-mediated incomplete trisomy rescue in micronuclei underlies various chromosomal rearrangements including SMCs, although other mechanisms such as U-type exchange may also yield SMCs. These assumptions are primarily based on observations of previously reported patients with complex rearrangements and our patient with a small SMC. Given the high frequency of trisomic cells in human preimplantation embryos, chromothripsis-mediated trisomy rescue may be a physiologically important phenomenon. Nevertheless, trisomy rescue has a potential to produce UPD, SMCs, and other chromosomal rearrangements. The concepts of trisomy rescue, chromothripsis, and micronuclei provide novel insights into the mechanism for the maintenance and modification of human chromosomes.

## Introduction

Small supernumerary marker chromosomes (SMCs) are defined as structurally abnormal chromosomes whose size is smaller than or equal to that of chromosome 20 on the same metaphase spreads (Liehr et al., 2011; Liehr, 2019). Small SMCs can have ring, centric minute, or inverted duplication structures and result in copy-number gain of the affected genomic regions (Liehr et al., 2011; Liehr, 2018; Liehr, 2019). Small SMCs can be present in both mosaic and non-mosaic statuses (Liehr et al., 2011; Al-Rikabi et al., 2018). Reportedly, ~70% of cases of small SMCs are *de novo* and are most frequently derived from chromosome 15 (Liehr, 2012).

Recent studies have suggested that *de novo* non-recurrent small SMCs result from incomplete trisomy rescue (Kurtas et al., 2019). Trisomy rescue is a physiological phenomenon to eliminate excessive chromosomes from trisomic cells (Fenech et al., 2011; Jones et al., 2012). When trisomy rescue is interrupted before complete elimination of target chromosomes, it may create various chromosomal rearrangements including small SMCs (Liehr, 2010; Kurtas et al., 2019). In this *Perspective* article, we discuss the underlying mechanisms and clinical significance of trisomy rescue in association with SMCs and other complex chromosomal rearrangements (CCRs). To this end, we introduce several patients, including our patient, who carried *de novo* CCRs.

## Trisomy Rescue Occurs in Micronuclei

Human preimplantation embryos frequently contain trisomic cells (Los et al., 2004; Liehr, 2010; Irani et al., 2018). Previous studies have suggested that ~1.7% of such trisomic cells have a chance to be corrected through trisomy rescue (Los et al., 2004; Irani et al., 2018). Trisomy rescue is assumed to take place predominantly between the first and fourth postzygotic cell divisions and result in a mosaic status of trisomic and disomic cells (Los et al., 1998; Liehr, 2010). Subsequently, the proportion of trisomic cells in the body would decrease over time because aneuploid cells are at a growth disadvantage compared to diploid cells. Thus, trisomic cells become undetectable in several postnatal clinical samples.

**Figure 1:**
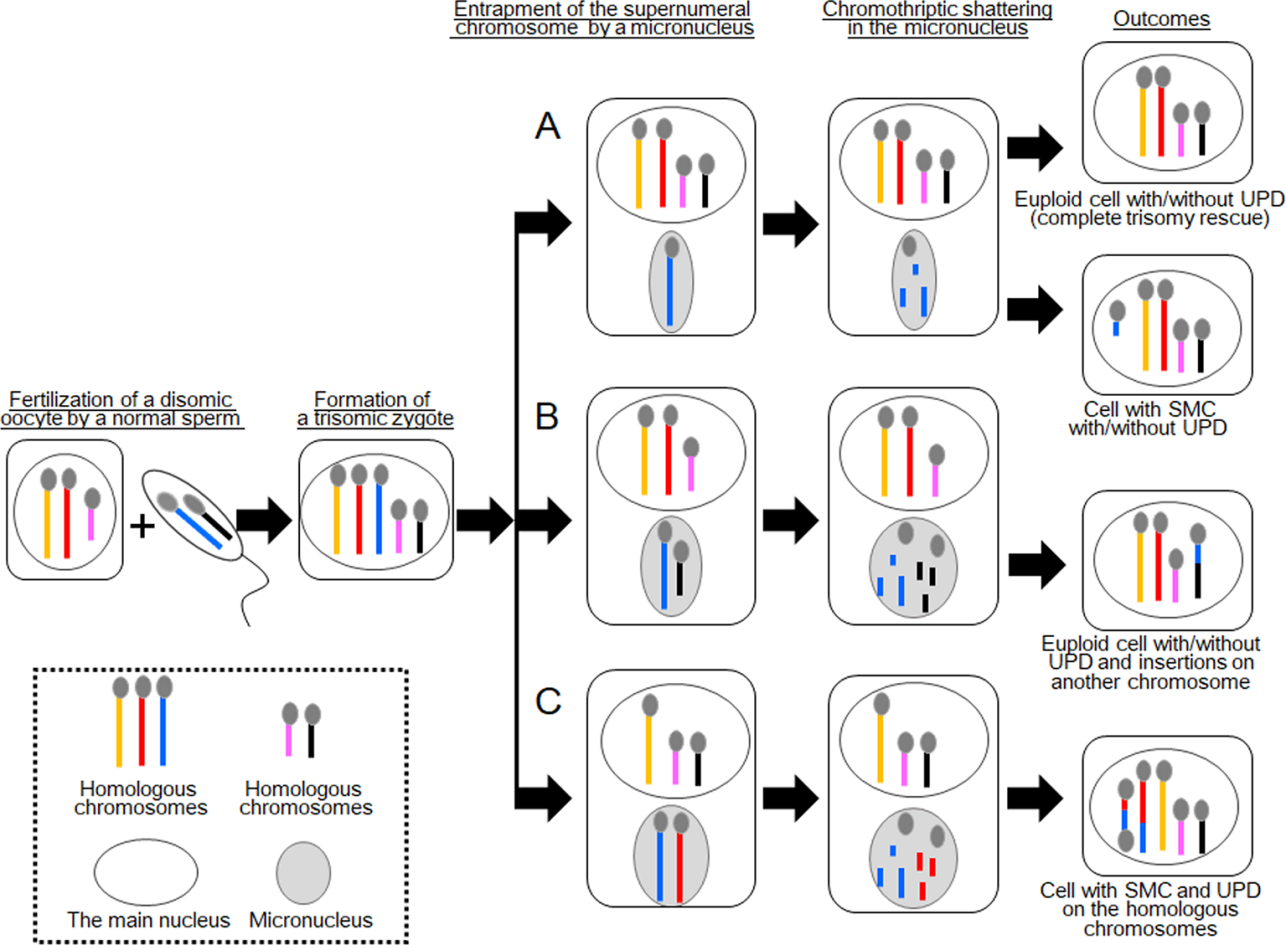
Schematic of the consequences of chromothripsis-mediated trisomy rescue in micronuclei. Two sets of homologous chromosomes are shown as yellow, red, and blue lines, and pink and black lines. During trisomy rescue, trisomic chromosomes (blue lines) are trapped into micronuclei (gray circles) and subjected to chromothriptic shattering. Micronuclei are capable of containing additional chromosomes. **(A)** Complete trisomy rescue creates a karyotypically normal cell, while partial trisomy rescue can generate a cell with a small supernumerary marker chromosome (SMC). One third of these cells develop uniparental disomy (UPD). **(B)** A normal chromosome (black line) may also be encapsulated in a micronucleus together with a supernumerary chromosome. In this case, partial trisomy rescue creates complex chromosomal rearrangements (CCRs) on multiple chromosomes, in addition to UPD. **(C)** Partial trisomy rescue can also produce SMC and UPD, when two homologous chromosomes (red and blue lines) are jointly trapped in a micronucleus.

Although trisomy rescue can normalize the number of chromosomes in aneuploid cells, 1/3 of the corrected cells exhibit uniparental disomy (UPD) (**Figure 1A**). For example, when a trisomic cell containing one paternally derived and two maternally derived chromosomes 15 is subjected to trisomy rescue and thereby loses the paternally derived chromosome 15, this cell develops maternal UPD(15). Indeed, trisomy rescue represents the major cause of UPD, although other mechanisms such as monosomy rescue and gamete complementation can also produce UPD (Shaffer et al., 2001). UPD caused by trisomy rescue is comprised of either complete heterodisomy or a combination of isodisomy and heterodisomy (Robinson et al., 1998; Kotzot and Utermann, 2005).

Trisomy rescue is assumed to occur in micronucleus, an aberrant cellular component frequently observed in cancer cells and, to a lesser extent, in normal cells (Zhang et al, 2015; Marcozzi et al., 2018). A micronucleus encapsulates one or a few chromosomes to separate them from the other chromosomes in the main nucleus (**Figure 1A**) (Zhang et al., 2015). Micronuclei have been implicated in the degradation of mis-segregated or supernumeral chromosomes (Jones et al., 2012; Zhang et al., 2015; Marcozzi et al., 2018). Jones et al. (2012) performed fluorescence *in situ* hybridization (FISH) analyses for micronuclei in leukocytes of 59 individuals, focusing on sex chromosome-containing micronuclei, and found that most micronuclei in young individuals are involved in trisomy rescue, whereas those in elder individuals are frequently associated with cellular errors leading to the degradation of an intact chromosome.

## Micronuclei are Known as the Platform for Chromothripsis

Micronuclei are known as the platform of chromothripsis, a recently discovered one-off cellular event that produces catastrophic structural changes exclusively on a single or a few chromosomes (Zhang et al., 2015). Chromothripsis was first discovered in the cancer genome and was soon after detected in the germline (Kloosterman et al., 2011; Liu et al., 2011). In addition, *de novo* occurrence of chromothripsis in postzygotic cells has also been documented (Kato et al., 2017). Constitutional chromothripsis usually creates CCRs with 3–20 breakpoints, while chromothripsis in cancer cells frequently generates rearrangements with more than 100 breakpoints (Liu et al., 2011). More recently, other one-off mutagenic events, such as chromoanasynthesis and chromoplexy, have been reported as underlying mechanisms for CCRs (Masset et al., 2016; Marcozzi et al., 2018). However, it remains to be clarified whether chromothripsis and these events are completely different phenomena. In this manuscript, we use the word “chromothripsis” to refer to all catastrophic cellular events leading to CCRs.

Presumably, chromothripsis in micronuclei follows the following processes (Poot and Haaf, 2015; Marcozzi et al., 2018); (i) a supernumeral or mis-segregated chromosome is encapsulated in a micronucleus, (ii) the trapped chromosome undergoes aberrant DNA replication and premature DNA condensation, (iii) the uncoordinated replication/condensation processes trigger DNA double-strand breaks and resultant chromosomal shattering, (iv) the contents of the micronucleus (DNA fragments) are incorporated into the main nucleus of a daughter cell, and (v) the fragments are randomly assembled and fused through non-homologous end joining and/or microhomology-mediated break-induced replication. Alternatively, when the components of the micronucleus fail to enter the main nucleus, these DNA fragments are degraded in the cytoplasm and lost.

Defective DNA replication in micronuclei may reflect their abnormal membrane structures (Holland and Cleveland, 2012). Specifically, the envelopes of micronuclei possess only a limited number of nuclear poles and, therefore, are predicted to provide an insufficient protein supply for DNA replication and repair (Holland and Cleveland, 2012). Moreover, micronuclei seem to be prone to irreversible nuclear envelope collapse during the interphase (Ly and Cleveland, 2017).

## Various Chromosomal Rearrangements can be Created in Micronuclei

Zhang et al. (2015) performed live-cell imaging and single-cell genome sequencing and elegantly confirmed that entrapment of one chromosome in a micronucleus can produce a spectrum of genomic rearrangements. Such rearrangements include catastrophic genomic crisis, as well as small circular chromosomes. Furthermore, because a micronucleus is capable of encapsulating more than one chromosome, a micronuclear chromothriptic event can create CCRs on multiple chromosomes (Storchová and Kloosterman, 2016).

In terms of trisomy rescue, micronucleus-mediated chromothripsis contributes to the elimination of excessive chromosomes to generate euploid cells (**Figure 1A**). However, when the process of chromothripsis is interrupted, it can cause partial chromosomal loss and/or rearrangements with or without UPD (**Figure 1A**) (Liehr, 2010). Co-occurrence of *de novo* CCRs and UPD, particularly those involving a set of homologous chromosomes, is indicative of incomplete trisomy rescue (Liehr et al., 2011; Kurtas et al., 2019). Previous studies have shown that a substantial proportion of UPD cases were accompanied by additional genomic or cytogenetic abnormalities (Liehr, 2010). Of these, SMCs accounted for ~4% of previously reported cases.

## Two Cases With CCRs Indicative of Partial Trisomy Rescue

In 2017, Kato et al. reported an interesting case with CCRs and UPD [case 1 in Kato et al. (2017)]. This case involved segmental UPD(4) and *de novo* CCRs on chromosome 14. The results of cytogenetic analysis and SNP genotyping suggested that this patient initially had trisomy 4 consisting of one paternally and two maternally derived chromosomes. Then, one of the maternally derived chromosomes 4 was fragmented into several pieces, some of which were inserted into the paternally derived chromosome 14 to create an interstitial deletion on the recipient chromosome. The remaining pieces of the maternal chromosome 4 seemed to be lost. All other autosomes and X chromosomes remained structurally intact. The most likely explanation for this case is that a cell with trisomy 4 went through partial trisomy rescue, during which one of the three chromosomes 4 and one normal chromosome 14 were trapped into a micronucleus and subjected to chromothripsis (**Figure 1B**). The chromosome 14 seems to have been accidentally trapped in the micronucleus because it was not associated with any numerical or structural abnormalities. The mutagenic processes appear to have occurred during early-postzygotic stages because both the maternally and paternally derived materials were involved in the CCRs, and this case showed no evidence of somatic mosaicism.

Furthermore, Kato et al. reported a case with CCRs and partial trisomy 5 [case 2 in Kato et al. (2017)]. This case comprised *de novo* CCRs on chromosomes 5 and 8 and a partial trisomy of chromosome 5. It appeared that chromosome 5 of the paternal origin was fragmented and some of the DNA fragments were inserted into the paternally derived chromosome 8, thereby deleting original sequences of that chromosome. Remarkably, the inserted fragments were derived from the nontransmitted chromosome 5 of the father. These results suggest that in this case, the zygote inherited two chromosomes 5 from the father and one from the mother. Subsequently, one of the two paternally derived chromosomes 5 was trapped into a micronucleus together with the paternally derived chromosome 8 and subjected to chromothripsis. The majority of the trapped chromosome 5 appeared to be lost during chromothripsis.

The results of these two cases imply that incomplete trisomy rescue can create *de novo* CCRs on multiple chromosomes, when one normal and one trisomic chromosomes are jointly incorporated into a micronucleus (**Figure 1B**). Such CCRs develop with or without UPD, as seen in cases 1 and 2, respectively. Considering that both cases 1 and 2 carried large insertions, micronucleus-mediated chromothripsis may favor amplification of genomic materials. Indeed, Kato et al. (2017) proposed that micronuclei formed from anaphase-lagging chromosomes may predispose a pulverized insertion.

## Our Case With UPD and a Small SMC

We recently encountered a patient with a small SMC, who possibly represents a novel case of atypical partial trisomy rescue. The patient was a 2-year-old boy with a 47,XY,+mar, ish dic(q13;q13)(D15Z1++, SNRPN++) karyotype. He presented with developmental delay, hypotonia, feeding difficulties, and epilepsy. We performed comparative genomic hybridization (CGH) using a catalog human CGH + single nucleotide polymorphism (SNP) array containing ~120,000 CGH probes and ~60,000 SNP probes for the entire genome (Sureprint G3; catalog number, G4890; 4 × 180 K format; Agilent Technologies, Palo Alto, CA, USA). The procedure was as described in the manufacturer’s instructions. The data were analyzed using the default settings of the Genomic Workbench Software (Version 6.5, Agilent Technologies). The copy-number of each genomic region was assessed from log2 ratios of the corresponding probes; log2 ratios between +0.4 and +0.6 and between +0.9 and +1.1 were considered the signs of 3-copy and 4-copy regions, respectively. Copy-number for the centromeric part of 15q11.2 could not be determined because of the presence of repetitive sequences. To determine the parental origin of chromosomal segments, we genotyped 20 microsatellite markers on chromosome 15 (**Supplementary Table 1**) using genomic DNA samples from the patient and his parents. PCR amplification was performed using fluorescently labeled forward primers and unlabeled reverse primers, as described previously (Matsubara et al., 2011). Subsequently, the PCR products were analyzed using the CEQ8000 sequencer (Beckman Coulter, Fullerton, CA, USA).

**Figure 2:**
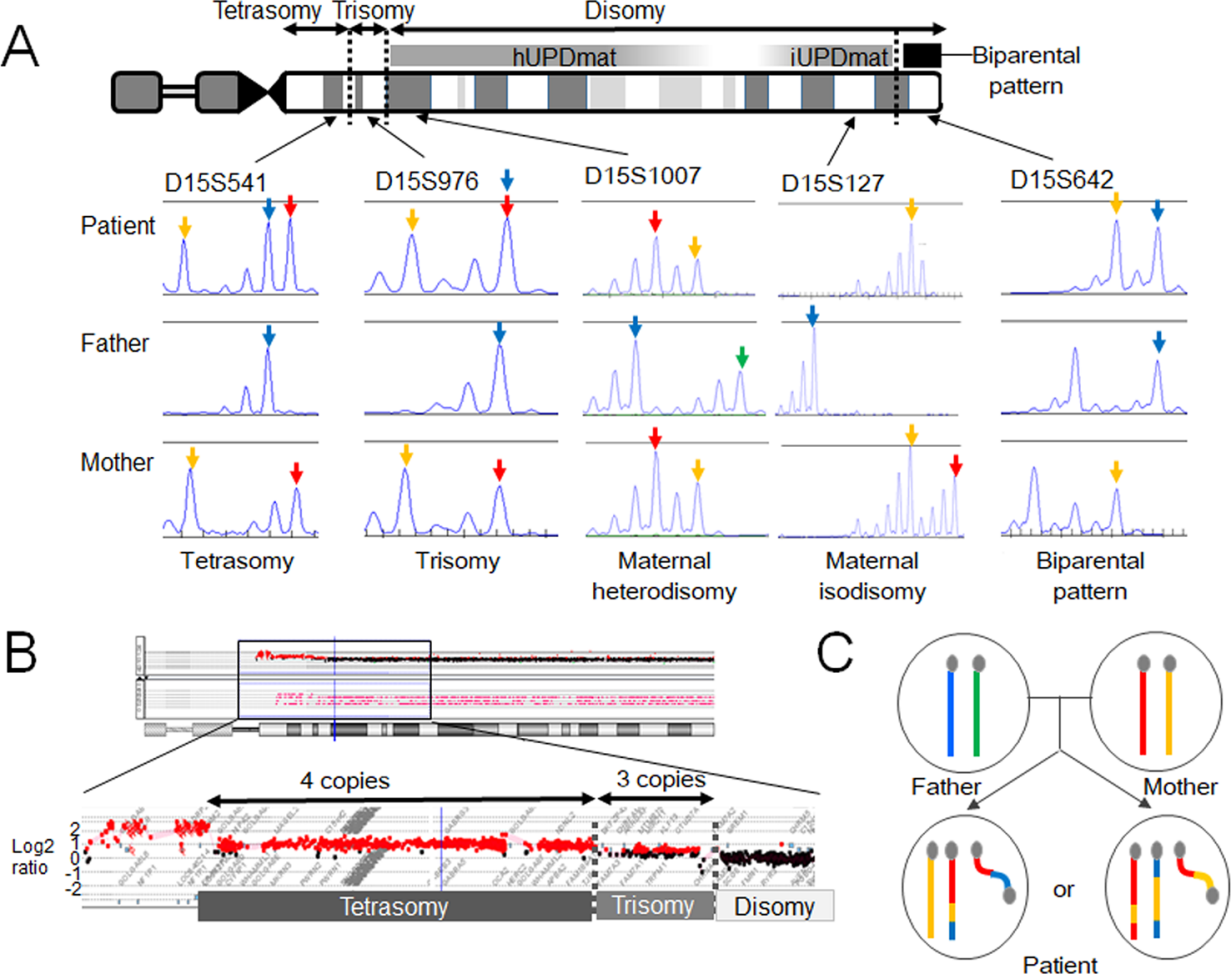
Molecular findings of our case. **(A)** Microsatellite analysis revealed segmental maternal disomy of chromosome 15. The red and yellow arrows depict alleles present in the mother, and the blue and green arrows indicate alleles present in the father. **(B)** Array-based comparative genomic hybridization detected copy-number gains in the 15q11-13 region. The breakpoints resided within low copy-number repeat regions. **(C)** Two most likely structures of chromosomes 15 in this case. One of the two structurally normal chromosomes 15 and the supernumerary marker chromosome are likely to comprise both maternal (red and yellow) and paternal (blue) materials. hUPDmat, maternal heterodisomy; iUPDmat, maternal isodisomy.

The results revealed that the boy harbors a *de novo* SMC comprising a dicentric chromosome 15, in addition to two structurally normal chromosomes 15 (**Figure 2**). The two chromosomes 15 exhibited maternal heterodisomy, except for the terminal region of the long arm which showed a biparental pattern (**Figure 2** and **Supplementary Table 1**). The SMC was an asymmetric dicentric chromosome containing both maternal and paternal materials. Collectively, this patient appeared to have a partial tetrasomy combined with segmental maternal UPD (15). No copy-number abnormalities were detected in the other chromosomes.

These findings indicate that this case initially had a trisomy due to the inheritance of two chromosomes 15 from the mother and one chromosome 15 from the father. We speculate that this individual have been subjected to chromothripsis-mediated partial trisomy rescue. If this is the case, a micronucleus encapsulated the paternally derived chromosome 15 and one of the two maternally derived chromosomes 15 (**Figure 1C**). Then, the trapped chromosomes were shattered and reassembled to form one structurally normal chromosome 15 and one SMC.

However, one may argue against chromothripsis having occurred in this case because the boy lacked chaotic rearrangements characteristic for chromothripsis. The other potential explanation of this case is U-type exchange (Liehr, 2019). It is possible that the genomic structure of this case has been generated through prezygotic or postzygotic U-type exchange and a subsequent homologous recombination (**Supplementary Figure 1**). Indeed, the breakpoints of this SMC resided within the 15q11–q13 region, a hotspot for homologous recombination (Pujana et al., 2002; Cassidy and Driscoll, 2009). Furthermore, previous studies have suggested that homologous recombination actually occurs in postzygotic cells, although its frequency is much lower than that in prezygotic cells (Kotzot, 2001; Roehl et al., 2010; Sasaki et al., 2013), and no studies have demonstrated that trisomic zygotes are associated with a particularly high frequency of postzygotic recombination. Thus, from this single case, we could not conclude that chromothripsis is actually involved in the development of small SMCs and UPD.

## Cases With SMCs Indicative of Chromothripsis

Recently, Kurtas et al. (2019) provided evidence that chromothripsis-mediated partial trisomy rescue accounts for certain percentage of the etiology of *de novo* small SMCs. The authors performed whole-genome sequencing and trio-genotyping for 12 cases with *de novo* small SMCs. The authors found that eight of the 12 individuals carried SMCs comprising non-contiguous portions of one chromosome, whereas the remaining four individuals had SMCs consisting solely of pericentromeric portions of a chromosome. In the eight patients, multiple portions of the SMCs were aligned in a disordered manner, suggesting that they have been subjected to chromothripsis. Moreover, these eight cases were likely to result from trisomy rescue because two of the eight cases showed a paternal origin of SMCs combined with maternal UPD of the homologous chromosomes, while the other six cases showed a maternal origin of the SMCs together with biparentally inherited homologous chromosomes. These findings indicate that the SMCs in the eight cases originated through maternal meiotic nondisjunction and subsequent chromothripsis-mediated degradation of excessive chromosomes. Indeed, most of these cases were associated with advanced maternal age, which is known as the major risk factor for meiotic nondisjunction (Jones, 2008). Al-Rikabi et al. (2018) also reported individuals who carried chromothripsis-consistent SMCs.

## Pathophysiological Significance of Trisomy Rescue in Human Embryos

Complete trisomy rescue in micronuclei increases the viability of human fetuses by reducing the negative effects of chromosomal aneuploidy. Considering the high frequency of trisomic cells in early-stage human embryos (Los et al., 2004; Liehr, 2010; Irani et al., 2018), trisomy rescue appears to be a physiologically important event. On the other hand, trisomy rescue frequently creates UPD. Although UPD is not necessarily pathogenic, it can cause imprinting disorders and unmask recessive mutations. It has been proposed that the frequency of UPD among newborns is as high as ~1/3,500 (Robinson, 2000; Yamazawa et al., 2010), and a substantial percentage of UPD cases result from trisomy rescue (Liehr, 2010; Liehr, 2019).

Moreover, partial trisomy rescue underlies various types of genomic rearrangements involving one or more chromosomes. For example, Bonaglia et al. (2018) demonstrated that the primary driver for *de novo* unbalanced translocations is maternal meiotic disjunction and subsequent partial trisomy rescue of the supernumerary chromosome. CCRs created by partial trisomy rescue often lead to congenital malformations and developmental delay by altering the copy-number or cis-regulatory machinery of the genes on the affected chromosomes (Poot and Haaf, 2015). Collectively, chromothripsis-mediated trisomy rescue in micronuclei appears to be a physiologically and pathologically important phenomenon.

## Conclusions

Trisomy rescue seems to be a physiologically indispensable event in human fetuses to reduce the deleterious effects of chromosomal aneuploidy. Nevertheless, trisomy rescue can produce UPD and genomic rearrangements. Recent studies, including our own, suggested that chromothripsis-mediated partial trisomy rescue is involved in the formation of small SMCs, although other mechanisms such as U-type exchange and postzygotic recombinations may also contribute to the development of such SMCs. The concepts of trisomy rescue, chromothripsis, and micronuclei provide novel insights into the origin of complex structural changes in the genome. Further studies on cases with various types of SMCs will clarify the molecular machineries involved in the maintenance and modification of chromosomal architectures.

## Data Availability Statement

The datasets generated for this study can be found in no repository but are available from the corresponding author upon reasonable request (no accession number).

## Ethics Statement

Our case study was approved by the Institutional Review Board Committee at the National Center for Child Health and Development. The guardian of the patient gave written informed consent in accordance with the Declaration of Helsinki for the participation in the study and the publication of the results.

## Author contributions

KM, MK, and MF contributed to study design. KM and MK performed molecular analysis. KY and TN performed clinical analysis. KM, MK, and MF contributed to the writing of the paper. All authors read and approved the final manuscript.

## Funding

This research was supported by the Grant-in-aid for Scientific Research on Innovative Areas (17H06428) from JSPS and grants from AMED, National Center for Child Health and Development, and Takeda Foundation. The funders had no role in the design of the study; the collection, analysis, or interpretation of data; or the writing of the manuscript.

## Conflict of Interest

The authors declare that the research was conducted in the absence of any commercial or financial relationships that could be construed as a potential conflict of interest.
