# Supplementary material for: De Novo Small Supernumerary Marker Chromosomes Arising From Partial Trisomy Rescue
Source: Front Genet. 2020 Feb 27;11:132. doi: 10.3389/fgene.2020.00132 (PMC7056893; doi:10.3389/fgene.2020.00132)
Supplement: Supplementary file 1 [file Image_1.pdf]

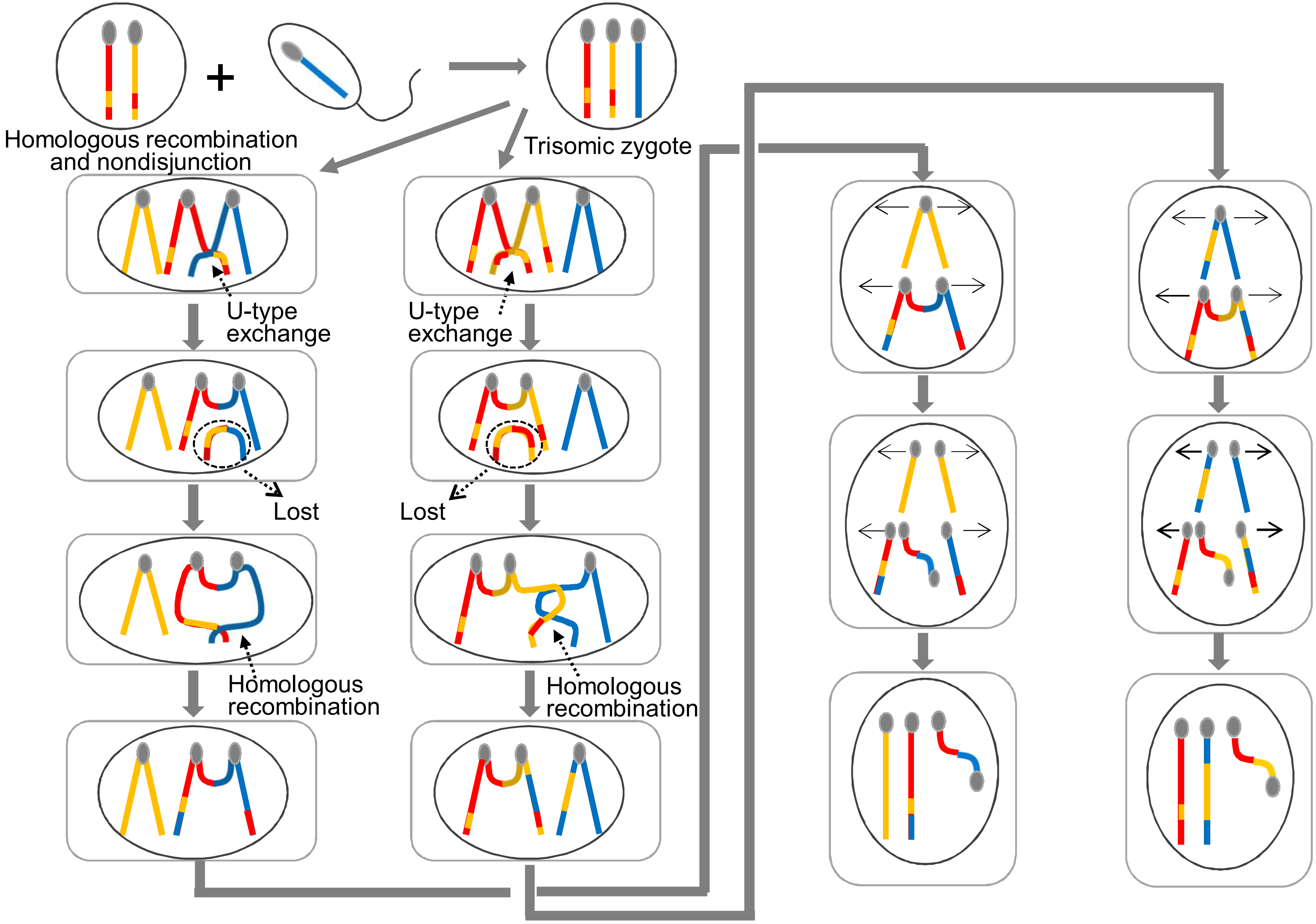

Supplementary Figure 1. Possible mechanisms of the small supernumerary marker chromosome in our case.
The small supernumerary marker chromosome (SMC) in our case can be explained by assuming that this embryo underwent meiotic homologous recombination, nondisjunction, prezygotic or postzygotic U-type exchange, and postzygotic homologous recombination. (The model of prezygotic U-type exchange is not shown in this figure.) It is also possible that the small SMC in this case was created through a chromothripsis-mediated mechanism depict in Figure 1C.
